# The Roles of Noradrenergic and Glucocorticoid Activation in the Development of Intrusive Memories

**DOI:** 10.1371/journal.pone.0062675

**Published:** 2013-04-29

**Authors:** Richard A. Bryant, Chloe McGrath, Kim L. Felmingham

**Affiliations:** 1 School of Psychology, University of New South Wales, Sydney, New South Wales, Australia; 2 School of Psychology, University of Tasmania, Hobart, Tasmania, Australia; Max Planck Institute of Psychiatry, Germany

## Abstract

Intrusive memories are a common feature of many psychological disorders. Recent evidence has potentially extended cognitive models of intrusions by identifying the role of biological markers of arousal at the time of consolidation in subsequent memory for emotional events. This study investigated the role of arousal during consolidation in the development of intrusive memories. Seventy-eight university students (37 men and 41 women) viewed 20 negative and 20 neutral images. Half the participants then underwent a cold pressor test (High Stress), immersing their hand in ice water, while the remaining participants immersed their hand in warm water (Low Stress). Samples of salivary alpha-amylase (sAA) and cortisol were collected from participants at baseline and following the stressor challenge. Participants completed a delayed free recall test and intrusion questionnaires two days later. Participants in the High Stress condition reported more intrusions of negative images than participants in the Low Stress condition. An interaction variable in a linear regression of increased noradrenergic and cortisol values predicted intrusive memories of emotional stimuli for men but not women. These findings are consistent with recent evidence of the combined effects of noradrenaline and corticoid responses to stress on emotional memories, and also with increasing evidence of gender differences in how stress hormones influence formation of emotional memories. These findings point to possible mechanisms by which development of intrusions may be prevented after consolidation of traumatic experiences.

## Introduction

Intrusive thoughts and memories are a common feature of many psychological disorders. Across disorders, intrusions share the common properties of occurring involuntarily, are typically vivid, negatively valenced, difficult to control, and interfere with ongoing cognitive functions [1]. Most models of intrusions recognize that arousal at the time of consolidation contributes to subsequent intrusions [1], [2]. These models propose divergent mechanisms that are associated with elevated arousal; including proposals that (a) encoded memories are not sufficiently embedded in one’s autobiographical memory base because of altered consolidation secondary to arousal [3], (b) events are encoded in fragmented and perceptually-based modes that lead to their subsequent intrusion into awareness [1], (c) memories are unintentionally activated by internal or external triggers that were initially conditioned with encoded memory [4], or (d) thoughts that are consistent with immediate and emotionally salient threats are more likely to intrude [5]. These models differ marginally on the emphasis they place on encoding and consolidation phases of memory formation, although major models posit that arousal during the consolidation process is pivotal in formation of intrusive memories because of the ongoing nature of arousal that typically occurs in the wake of an aversive experience [6]. Despite this convergence, there is a dearth of evidence for the role of arousal in intrusive memories.

Although there is little evidence concerning the role of arousal in development of intrusive memories, there is much evidence that arousal leads to better intentionally recalled emotional memories. For example, noradrenergic activation at the time of encoding/consolidation leads to stronger intentional retrieval for emotional events [7]. Specifically, administration of adrenergic receptor blockers (e.g., propranolol) decreases memory for emotional events relative to placebo [8]. Administration of propranolol reduces activation in the amygdala, as well as memory for emotional (but not neutral) material [9]. Further, memory for emotional, but not neutral, stimuli is associated with endogenous noradrenergic activation at the time of encoding [10].

Glucocorticoids have also been implicated in the modulation of emotional memories. For example, there is evidence that administration of hydrocortisone results in superior recall of subsequently presented emotional (rather than neutral) stimuli [11]. Increasing evidence suggests that the *interaction* of noradrenergic and glucocorticoid systems may underlie the superior recall of emotional memories [7]. It is proposed that glucocorticoid activation facilitates the noradrenergic cascade in the basolateral nucleus of the amygdala (BLA), thereby enhancing retention of emotional stimuli. This is supported by evidence that infusion of glucocorticoid antagonists into the BLA reduces the effects of β-adrenoceptor agonist on emotional memory [12].

Although the roles of adrenergic and glucocorticoid systems have been studied in the context of intentionally retrieved memories, these systems have not been investigated in terms of intrusive memories. Although intrusive memories are more likely to occur when the memory is emotionally charged [13], intrusive memories may function differently from intentionally retrieved memories. Accordingly, the goal of this project was to investigate the roles of noradrenergic and glucocorticoid activation in response to a stressor on subsequent intrusive memories of emotional events. Specifically, we extended on a previous study of emotional memory [14] by presenting participants with neutral and aversive images and subsequently required half of participants to immerse a hand in icy water; intrusive memories were subsequently assessed two days later. We manipulated arousal during consolidation because theories of intrusions suggest that arousal in the consolidation phase is pivotal in formation of intrusive memories [6].

Potential sex differences may exist in intrusive memories in men and women because of evidence that women have stronger retention for emotional memories [14], [15]. Further, the role of glucocorticoids in intrusions may differ across gender [16]; further, there is evidence that the influence of cortisol on subsequent memory for emotional events is dependent on menstrual phase [17] [18]. There is evidence of differential glucocorticoid release across gender ([19], [20]. Accordingly, we tested equal numbers of males and females to test for differential patterns of response. We hypothesized that intrusions would be associated with (a) emotional more than neutral memories, (b) under high rather than low stress, and (c) would be predicted by noradrenergic and/or cortisol increase.

## Materials and Methods

### Participants

Thirty-seven male and 41 female first year psychology students of mean age 19.78 years (*SD* = 2.49) at the University of New South Wales were recruited in return for course credit. Screening for potential participants restricted female participants to those who were not using oral contraceptives in order to limit hormonal variation.

### Stimuli

Stimuli comprised 40 color photographs selected from the International Affective Picture System (IAPS; [21]), comprising 20 High Stress/negative images (mean arousal rating: 6.13, mean valence rating: 4.96) and 20 Low Stress/neutral images (mean arousal rating: 2.66, mean valence rating: 2.85).

### Procedure

Participants were instructed to refrain from exercising the day before, eating one hour before, and drinking caffeine or alcohol three hours before the experimental session that occurred between 12∶00 and 17∶30 to prevent confounding salivary measures [22]. Following written informed consent, participants were asked to drink water at the beginning of the experiment, and a saliva initial sample was taken 15 minutes afterwards. Participants then viewed the images in a random order. Participants viewed each picture for five seconds and rated each picture along dimensions of pleasantness (1 = *very pleasant*, 5 = *very unpleasant*) and emotional arousal (1 = *extreme emotional arousal*, 5 = *no emotional arousal*) immediately after viewing each picture.

Participants then immersed their dominant hand above the wrist in water for three minutes; for participants in the High Stress condition the ice water was 0° Celsius), whereas for those in the Low Stress condition the water was 30–40 degrees Celsius. After the three minutes, a second sAA sample was immediately collected. Participants were then administered the Depression, Anxiety, Stress Scale 21 (DASS-21) to assess depression and anxiety levels. Participants then completed a time-filling task (crossing out the letter “e” in two pages of text) to allow for a 15-minute delay before obtaining a second cortisol sample.

The participants returned two days later and completed a surprise free-recall memory test of the initially presented images by asking them to write detailed descriptions of any mages from the initial session. Participants then completed a questionnaire assessing intrusive images of the presented images. Participants were instructed to “*Think about the pictures you were shown when you came to the experiment a few days ago”, and answer each of the three following questions about any memories of these images*”. Three items were selected from the Intrusion subscale of the Impact of Event Scale (IES; [23]) that most closely measure the construct of intrusive memories (Item 1: *any reminder brought back feelings of it*, Item 4: *I thought about it when I didn’t mean to*, and Item 5: *pictures about it popped into my mind*). Participants answer each of the three questions in relation to any negative images and separately for any neutral images (in a counter-balanced order) on a 5-point Likert scale (0 = *not at all*, 4 = *extremely*).

### Salivary Analyses

Saliva samples were acquired via cotton salivettes and were immediately stored frozen at −20°C until assay. Saliva samples were centrifuged at 1500 g for 15 minutes and sAA and salivary cortisol levels were assessed using Salimetrics (State College, PA) cortisol ELISA kits. Saliva samples were centrifuged at 1500 g for 15 minutes and assessed using standardized assays (Salimetrics, State College, PA) and salivary cortisol was assessed using Salimetrics cortisol ELISA kits. Salivary alpha-amylase is a marker of norepinephrine [24], and there is evidence that the salivary enzyme is a superior assessment of central endogenous noradrenergic activation compared to measurement of norepinephrine via blood plasma [25]. The percentage of variability within and between the sAA assays was 4.4% and 9.3%, respectively. The percentage of variability within and between the cortisol assays was 3.5% and 5.05%, respectively.

## Results

### Participant Characteristics

Mean participant characteristics are presented in Table 1. Separate 2 (Condition) ×2 (Gender) analyses of variance (ANOVA) of participants’ ages, and DASS scores indicated no significant main or interaction effects, suggesting no systematic baseline differences between groups.

**Table 1:** Participant Characteristics and Stress Responses.

	High Stress		Low Stress	
	Male	Female	Male	Female
Age	20.33 (3.22)	19.50 (1.73)	19.74 (3.02)	19.10 (1.88)
DASS-Depression	4.33 (3.90)	3.35 (2.85)	4.05 (3.64)	4.63 (3.84)
DASS-Anxiety	2.83 (2.18)	2.35 (2.06)	1.95 (1.95)	3.10 (2.60)
DASS-Stress	5.44 (3.65)	4.90 (3.18)	4.47 (3.56)	5.89 (3.94)
Neutral Arousal Rating	4.63 (.72)	4.39 (.78)	4.20 (.88)	4.37 (.75)
Negative Arousal Rating	3.02 (.77)	2.85 (.97)	2.85 (.94)	2.68 (.84)
Neutral Valence Rating	3.13 (.39)	4.17 (.53)	2.94 (.18)	2.98 (.37)
Negative Valence Rating	4.07 (.46)	4.17 (.53)	4.09 (.43)	4.11 (.58)
sAA Baseline	119.13 (82.26)	115.40 (78.64)	105.98 (71.82)	111.42 (63.13)
sAA Post Stress	98.20 (60.31)	96.01 (37.41)	86.71 (68.85)	95.26 (65.19)
Cortisol Baseline	18.11 (17.98)	13.53 (15.19)	15.15 (20.47)	13.34 (14.56)
Cortisol Post Stress	32.80 (29.48)	30.82 (46.74)	12.23 (12.21)	8.45 (7.25)

*Note.* Standard deviations appear in parentheses. sAA measured in μ/ml. Cortisol measured in nmol/dl.

### Manipulation Check

**Figure 1:**
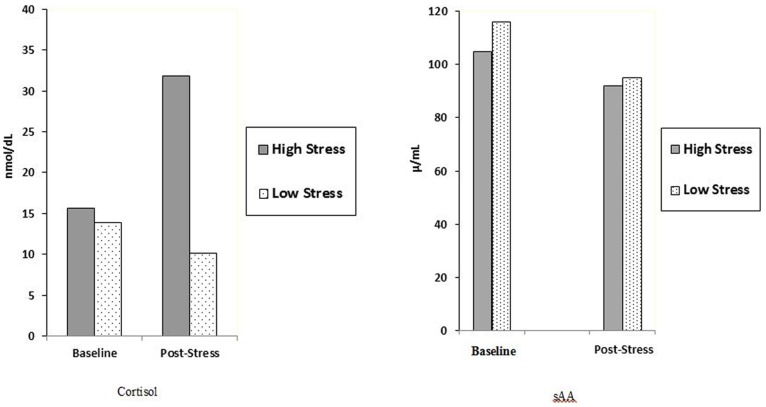
Average salivary alpha amylase and cortisol levels at each experimental phase.

sAA and cortisol levels are reported in Figure 1 (see also Table 1). A 2 (Condition) ×2 (Gender) ×4 (Assessment Point) ANOVA of sAA increase indicated a significant main effect for Assessment Point, *F* (3, 70) = 4.88, *p = *.004. Specifically, participants’ sAA levels decreased significantly from watching the images to after immersing their hands in water (regardless of stress condition). A 2 (Condition) ×2 (Gender) ×3 (Assessment Point) ANOVA of cortisol increase indicated a significant main effect for Condition, *F* (2, 72) = 5.01, *p = *.01. Participants in the High Stress Condition displayed higher cortisol levels than those in Low Stress condition following the stressor manipulation. That is, participants in the High Stress condition experienced a greater increase in cortisol than those in the Low Stress condition immediately following the stress manipulation (*p = *.001).

### Free Recall

Mean recall scores are reported in Table 2. A 2 (Condition) ×2 (Gender) ×2 (Valence) ANOVA of the free recall responses indicated a significant effect for Valence [*F*(1,74) = 130.74, *p*<0.000, η = .65], and a significant Condition × Gender × Valence interaction effect [*F*(1,72) = 5.29, *p*<0.05, η = .07] (see Table 1). Participants reported more negative than neutral memories. To interpret the three-way interaction, two separate Condition × Gender ANOVAs were conducted for negative and neutral memories, respectively. There was a significant Condition × Gender interaction effect for negative memories [*F*(1,72) = 9.41, *p* = . 0.003, η = .12]; women in the High Stress condition recalled more negative memories than women in the Low Stress condition [*F*(1,37) = 4.08, *p* = . 0.000].

**Table 2:** Free Recall and Intrusive Memory Responses.

	High Stress		Low Stress			
	Male	Female	Male	Female	*F*	*p*
**Neutral Free Recall**	2.22 (1.89)	2.85 (1.35)	2.32 (1.41)	1.94 (1.61)	0.56	.46
**Negative Free Recall**	5.28 (3.30)	6.90 (2.44)	5.94 (2.29)	4.00 (1.94)	4.67	.01
**Neutral Intrusions**	0.39 (0.85)	1.00 (1.62)	1.05 (1.47)	1.26 (2.13)	1.31	.25
**Negative Intrusions**	2.33 (2.22)	2.70 (258)	2.80 (2.50)	1.58 (2.52)	0.02	.89

*Note.* Standard deviations appear in parentheses.

### Intrusions

Mean intrusions scores are reported in Table 2. A 2 (Condition) × 2 (Gender) × 2 (Valence) ANOVA of the intrusion questionnaire scores indicated a significant effect for Valence [*F*(1,72) = 17.18, *p*<0.000, η = .19], and a significant Valence × Condition interaction effect [*F*(1,72) = 4.91, *p*<0.05, η = .06] (see Table 1). Participants reported more intrusions of the negative compared to the neutral pictures. Furthermore, participants in the High Stress condition reported more intrusions of the negative pictures than participants in the Low Stress condition (*p*<.005).

### Relationship between Free Recall and Intrusions

To determine the relationship between recall responses and subsequent intrusions, we calculated Pearson correlation coefficients between these responses for men and women respectively (on the basis that differential retrieval patterns were observed across genders). In terms of men, free recall of neutral images was positively correlated with subsequent intrusions of neutral intrusions (*r = *.34, *p = *. 04). Conversely, women displayed a significant correlation between free recall of negative images and subsequent intrusions of negative intrusions (*r = *33, *p = *. 04).

### Role of Noradrenergic Activation and Cortisol

To index the contribution of noradrenergic and cortisol activation following the cold pressor test/control exercise on subsequent recall and intrusions, we conducted separate linear regressions to predict recall and intrusions of the negative and neutral images, respectively. On the basis that these hormones function differentially in men and women, we conducted these regressions separately for male and female participants. Arousal manipulation condition was entered at Step 1, z-transformed values of sAA increase from baseline to following the arousal manipulation was entered at Step 2, z-transformed values of cortisol increase from baseline to following the arousal manipulation was entered at Step 3, and the interaction between the latter two variables was entered at Step 4. In terms of recall of images, being administered the cold water manipulation predicted negative recall in women, accounting for 32% of the variance (see Table 3). Cortisol increase after the arousal manipulation predicted better recall of neutral memories in men accounting for 21% of the variance (see Table 4). In terms of negative intrusions for males, the only significant predictor was the interaction variable of increased cortisol and sAA, accounting for 16% of the variance; there were no significant predictors of negative intrusions for females (see Table 5). There were no significant predictors for intrusions of neutral images (Table 6).

**Table 3:** Summary of Hierarchical Regression Models for Recall of Negative Images.

	*B*	SEB	*β*	*p*
***Males***				
**Step 1: Arousal** **condition**	−1.13	1.02	−.20	.27
**Step 2: sAA Increase**	.28	.98	.05	.78
**Step 3: Cortisol** **Increase**	.61	.50	.25	.23
**Step 4: sAA** ×**Cortisol Increase**	−.36	1.27	−.06	.78
***Females***				
**Step 1: Arousal** **condition**	2.67	.77	.51	.001
**Step 2: sAA Increase**	.05	.37	.02	.88
**Step 3: Cortisol** **Increase**	.50	.34	.21	.15
**Step 4: sAA** ×**Cortisol Increase**	−.19	.45	−.06	.68

*Note.* Males: Step 1 R^2^ = .01, Δ R^2^ = .01. Step 2 R^2^ = .02, Δ R^2^ = .00. Step 3 R^2^ = .09, Δ R^2^ = .07, Step 4 R^2^ = .09, Δ R^2^ = .00.

Females: Step 1 R^2^ = .31, Δ R^2^ = .32. Step 2 R^2^ = .29, Δ R^2^ = .00. Step 3 R^2^ = .31, Δ R^2^ = .04. Step 4 R^2^ = .29, Δ R^2^ = .00.

**Table 4:** Summary of Hierarchical Regression Models for Recall of Neutral Images.

	*B*	SEB	*β*	*p*
***Males***				
**Step 1: Arousal** **condition**	−.23	.52	−.07	.66
**Step 2: sAA Increase**	−1.01	.49	−.31	.07
**Step 3: Cortisol** **Increase**	.82	.26	.57	.003
**Step 4: sAA** ×**Cortisol Increase**	.74	.64	.21	.26
***Females***				
**Step 1: Arousal** **condition**	.83	.48	.27	.09
**Step 2: sAA Increase**	−.43	.23	−.27	.08
**Step 3: Cortisol** **Increase**	.22	.21	.16	.31
**Step 4: sAA** ×**Cortisol Increase**	.40	.28	.21	.17

*Note*. Males: Step 1 R^2^ = −.03, Δ R^2^ = .00. Step 2 R^2^ = .00, Δ R^2^ = .06. Step 3 R^2^ = .20, Δ R^2^ = .21. Step 4 R^2^ = .21, Δ R^2^ = .03.

Females: Step 1 R^2^ = .08, Δ R^2^ = 10. Step 2 R^2^ = .14, Δ R^2^ = .08. Step 3 R^2^ = .14, Δ R^2^ = .02. Step 4 R^2^ = .17, Δ R^2^ = .04.

**Table 5:** Summary of Hierarchical Regression Models for Intrusions of Negative Images.

	*B*	SEB	*β*	*p*
***Males***				
**Step 1: Arousal** **condition**	−.01	.71	−.00	.99
**Step 2: sAA Increase**	−.23	.60	−.06	.71
**Step 3: Cortisol** **Increase**	.54	.46	.30	.25
**Step 4: sAA** ×**Cortisol Increase**	1.88	.73	.65	.01
***Females***				
**Step 1: Arousal** **condition**	1.20	.88	.23	.18
**Step 2: sAA Increase**	.51	.55	.15	.36
**Step 3: Cortisol** **Increase**	−.31	.44	.13	.48
**Step 4: sAA** ×**Cortisol Increase**	−.12	.94	−.02	.90

*Note*. Males: Step 1 R^2^ = .01, Δ R^2^ = .01. Step 2 R^2^ = .03, Δ R^2^ = .01. Step 3 R^2^ = .02, Δ R^2^ = .04, Step 4 R^2^ = .13, Δ R^2^ = .16.

Females: Step 1 R^2^ = .02, Δ R^2^ = .04. Step 2 R^2^ = .02, Δ R^2^ = .03. Step 3 R^2^ = .01, Δ R^2^ = .01. Step 4 R^2^ = .01, Δ R^2^ = .00.

**Table 6:** Summary of Hierarchical Regression Models for Intrusions of Neutral Images.

	*B*	SEB	*β*	*p*
***Males***				
**Step 1: Arousal**	−.48	.44	−.20	.99
**Step 2: sAA Increase**	−.70	.37	−.30	.71
**Step 3: Cortisol** **Increase**	−.21	.28	−.19	.25
**Step 4: sAA** ×**Cortisol Increase**	−.27	.44	−.16	.01
***Females***				
**Step 1: Arousal** **condition**	−.06	.74	−.01	.93
**Step 2: sAA Increase**	−.27	.46	−.10	.57
**Step 3: Cortisol** **Increase**	−.21	.38	−.11	.58
**Step 4: sAA** ×**Cortisol Increase**	−.70	.79	−.17	.38

*Note*. Males: Step 1 R^2^ = .05, Δ R^2^ = .07. Step 2 R^2^ = .11, Δ R^2^ = .09. Step 3 R^2^ = .09, Δ R^2^ = .00, Step 4 R^2^ = .08, Δ R^2^ = .01.

Females: Step 1 R^2^ = −.02, Δ R^2^ = .00. Step 2 R^2^ = −.04, Δ R^2^ = .00. Step 3 R^2^ = .−.07, Δ R^2^ = .00. Step 4 R^2^ = −.07, Δ R^2^ = .02.

## Discussion

The focus of this study was on the occurrence of intrusive memories. In accord with one previous finding, we noted that intrusive memories were more likely to occur following consolidation of negative, rather than neutral, stimuli [13]. Importantly, we found this pattern was greater when participants experienced stress (via immersing their hand in icy water) during the consolidation phase. This interaction suggests that the level of stress attached to an emotional event at the time of consolidation contributes to the subsequent occurrence of intrusions. This finding provides support for prevailing models of intrusive memories that posit that the arousal at the time of aversive experiences results in enhanced consolidation of these memories, which may contribute to these memories intrusively and unintentionally being retrieved [1], [2].

Reinforcing the conclusion that stress plays a critical role in the development of intrusive memories, we found that the variable in the regression analysis that accommodated noradrenergic and glucocorticoid activations significantly predicted subsequent intrusions, over the effect of the stress manipulation. This finding is consistent with increasing evidence that it is the *combined* increase of noradrenaline and corticosteroid hormones that influence memory for emotional events [12], [26]. It also accords with findings of strongest amygdala recruitment when combining yohimbine and hydrocortisone [27], as well as the interactive effect of these agents on hippocampal activation during encoding of emotional material ([28]. It is proposed that glucocorticoids pass the blood-brain barrier readily, and thereby facilitate noradrenaline-mediated effects in the amygdala; this interaction of both glucocorticoid and noradrenergic activation may therefore contribute to the occurrence of subsequent intrusive memories of emotional experiences. The observation that negative intrusions were predicted by noradrenergic/glucocorticoid increase, but not by the stress manipulation alone, suggests that the individual variability in how participants respond to external stressors (such as cold water) plays an important role in the extent to which the stress response results in subsequent intrusive memories.

The observation of interacting influence of glucocorticoid and noradrenergic activation on subsequent intrusive memories in men but not women accords with previous findings of gender differences in associations between memory performance and stress hormones [29], [30]. For example, it has been reported that whereas an association between cortisol increase and emotional memory performance is observed in males, it is not found in females [16]. These relationships may not have been found in females because of variation of ovarian hormonal levels throughout the menstrual cycle. One study found that cortisol level and memory retention was correlated in females who encoded during the mid-luteal phase but did not for females in other phases of the menstrual cycle ([17]. There is also evidence that women in the luteal phase have more intrusive memories compared to women in the follicular phase [13], and female trauma survivors are more likely to experience flashback memories if they experience the trauma in the mid-luteal phase [31]. One limitation of this study is that we did not control for menstrual cycle in this study, and future studies need to assess estrogen and progesterone to determine the extent to which sex hormones moderate the relationship between stress and subsequent intrusions.

Consistent with previous research, we found superior free recall of negative relative to neutral information [32], [33]. This pattern was observed particularly in women who were exposed to High Stress at the time of memory consolidation. This pattern is also consistent with growing evidence of superior emotional memory in women, and the role of both stress and sex hormones in mediating emotional recall [15]. In men, the increase in cortisol following the arousal manipulation predicted better recall of neutral images. This observation is consistent with evidence in animals and humans that glucocorticoid increase results in enhanced learning and memory across a range of tasks [16], [34], [35]. It is unclear why this effect was not evident for negative images but it is possible that the aversive nature of these images resulted in less variance of their recall. One unexpected finding was that men did not display enhanced recall after being exposed to the stressor; this is inconsistent with a number of findings of male participants displaying stress-related enhancement of emotional memories [16], [36], [37]. One cannot attribute this finding to a lack of cortisol increase in men because they display a marked increase in cortisol following the cold water manipulation. One possible explanation is that the men in this study had elevated memory for the negative images in the warm water condition, with significantly greater recall of these images than women [*t*(38) = 2.61, *p = *.01]. The elevated recall of negative stimuli for men in the non-stressful condition may have reduced the possibility of a difference in recall scores between the stress and control conditions.

Even though we found that women who experienced arousal during the consolidation phase had greater memory for emotional events than those who did not, this difference was not observed in the frequency of subsequent intrusions. This discrepancy suggests that the patterns observed in recall of emotional memories are not linearly related to those observed in intrusive memories of the same events. This conclusion is underscored by the modest correlations between free recall and intrusions (*r* = .34 for men in relation to neutral stimuli and *r = *.33 for women in relation to emotional stimuli). It should be noted that whereas some models of intrusions emphasize the role of arousal during encoding and consolidation ([1], [2], others highlight the role of processes that occur following consolidation, including suppression to avoid the memory [38], or subsequent focus on memories arising from the need to explain incongruent experiences [39].

We did not comprehensively index other factors that occurred following consolidation, such as attempts to suppress the memories or attributions made about the memories, which may influence the occurrence of intrusive memories. Future research needs to pay more attention to the different processes that underpin consolidation of emotional memories that are intentionally and unintentionally retrieved. We also note that enhanced corticoids at the time of retrieval generally impair memory performance ([40]; we did not index sAA or cortisol over the following days after viewing the images and so we cannot determine the role of stress hormones at retrieval on intrusive memories.

We recognize that sAA did not increase as a function of either watching the negative images or immersing their hands in the icy water. This suggests that our stress manipulation may not have been sufficient, even though we did observe a marked cortisol increase in participants in the High Stress condition following the stress manipulation. We note that whereas we required participants to immerse their hands in the icy water, other studies have required participants to immerse their arms in the water [41], and even combined this with an additional social stressor, to enhance the stress manipulation [42]. Replication of this study should index the occurrence of intrusions following more stressful inductions. Further, we note we did not conduct a test for psychoactive drugs, which can influence both salivary response and memory capability.

In summary, this study provides the first evidence of the role of stress hormones at the time of consolidation on development of subsequent intrusive memories. This finding accords with evidence of the role of the interactive influences of noradrenergic and glucocorticoid activation at the time of encoding on emotional memories. Considering that intrusive memories are conceptualized as key to many psychological disorders, attempts at attenuating the roles of noradrenergic and/or glucocorticoid response during memory consolidation may contribute to prevention of adverse psychological states. Further study is required that delineates the direct interaction of noradrenergic and glucocorticoid activation, rather than relying on inferences from regression analyses, to clarify the impact of these systems on intrusive memories.
